# Historical factors that have shaped the evolution of tropical reef fishes: a review of phylogenies, biogeography, and remaining questions

**DOI:** 10.3389/fgene.2014.00394

**Published:** 2014-11-13

**Authors:** Peter F. Cowman

**Affiliations:** Department of Ecology and Evolutionary Biology, Yale UniversityNew Haven, CT, USA

**Keywords:** coral reef fishes, ancestral biogeography, marine tropics, phylogeny, diversification

## Abstract

Biodiversity patterns across the marine tropics have intrigued evolutionary biologists and ecologists alike. Tropical coral reefs host 1/3 of all marine species of fish on 0.1% of the ocean’s surface. Yet our understanding of how mechanistic processes have underpinned the generation of this diversity is limited. However, it has become clear that the biogeographic history of the marine tropics has played an important role in shaping the diversity of tropical reef fishes we see today. In the last decade, molecular phylogenies and age estimation techniques have provided a temporal framework in which the ancestral biogeographic origins of reef fish lineages have been inferred, but few have included fully sampled phylogenies or made inferences at a global scale. We are currently at a point where new sequencing technologies are accelerating the reconstruction and the resolution of the Fish Tree of Life. How will a complete phylogeny of fishes benefit the study of biodiversity in the tropics? Here, I review the literature concerning the evolutionary history of reef-associated fishes from a biogeographic perspective. I summarize the major biogeographic and climatic events over the last 65 million years that have regionalized the tropical marine belt and what effect they have had on the molecular record of fishes and global biodiversity patterns. By examining recent phylogenetic trees of major reef associated groups, I identify gaps to be filled in order to obtain a clearer picture of the origins of coral reef fish assemblages. Finally, I discuss questions that remain to be answered and new approaches to uncover the mechanistic processes that underpin the evolution of biodiversity on coral reefs.

## INTRODUCTION

**FIGURE 1:**
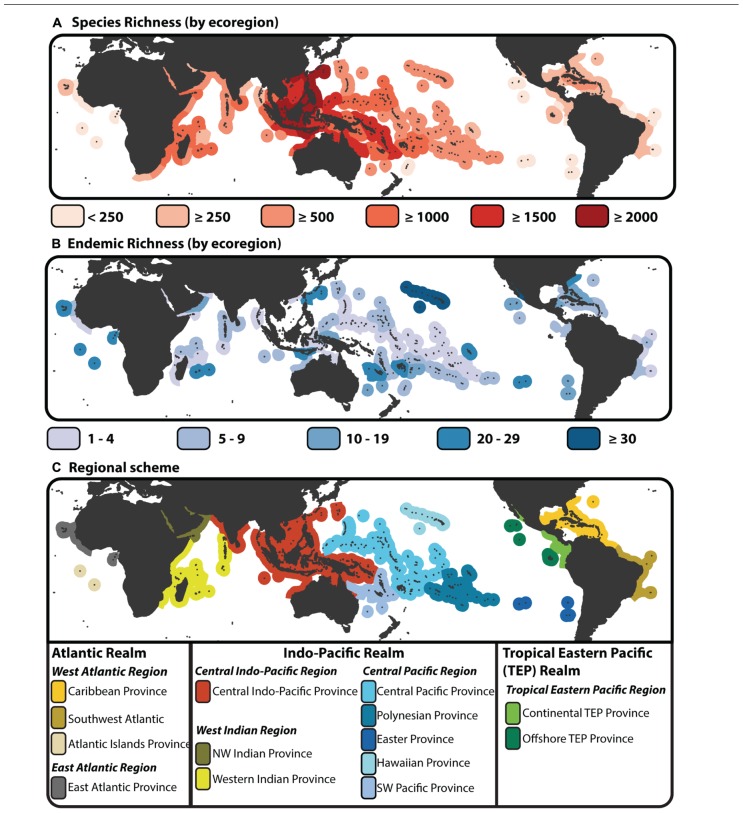
**Species richness, endemism and provinciality of tropical reef fishes. (A)** Map of species biodiversity by tropical ecoregion (Spalding et al., 2007) with color gradient denoting areas of high species richness (dark red) to areas of low species richness (light red). **(B)** Map of endemic species by ecoregion. Under this scheme a species is endemic if it is only found in a single ecoregion, i.e., a regional assessment of endemism rather that designated by percent of area comparison (Hughes et al., 2002). Species richness and endemic estimates are based on species counts from the “checklist” × “all species” dataset of Kulbicki et al. (2013). **(C)** Biogeographic delineation of tropical Realms, Regions, and Provinces based on species dissimilarity analysis of Kulbicki et al. (2013). This biogeographic scheme is base on checklists as base units (see Kulbicki et al., 2013), however here the scheme is imposed onto the tropical ecoregions of Spalding et al. (2007).

A latitudinal gradient in species diversity is a common feature of many taxonomic groups, both terrestrial and marine (Willig et al., 2003; Hillebrand, 2004). However, a longitudinal gradient in species diversity is also apparent across the marine tropics. Fishes exemplify this diversity gradient (Hughes et al., 2002; Tittensor et al., 2010) driven largely by patterns of species richness associated with tropical coral reef habitats. Species richness of reef associated fishes forms an enigmatic “bullseye” pattern centered on the Indo-Australian Archipelago (IAA; **Figure 1A**). This region has also been called several other names (reviewed by Hoeksema, 2007), but its position at the center of this species richness gradient has given it status as the largest marine biodiversity hotspot, covering two thirds of the global equatorial tropics (Bellwood et al., 2012). Unlike terrestrial biodiversity hotspots (Myers, 1988; Myers et al., 2000), centers of endemism are not concordant with the center of highest species diversity, whether endemic species are defined by regional checklists (**Figure 1B**), or the extent of their geographic range (Hughes et al., 2002; but see Mora et al., 2003). Traditional hotspot analysis of the marine environment has identified endemic centers under high levels of threat (Roberts et al., 2008), however these 10 defined areas of endemism exclude some areas that have the high diversity of overlapping, wide ranging species. In addition to the distinctive biodiversity gradient, the tropics have been divided into a number of realms, regions, provinces and eco-regions based on shared environmental characteristics (Spalding et al., 2007), composition of endemic taxa (Briggs and Bowen, 2012), or measures of species dissimilarity (Kulbicki et al., 2013). Although these differing regional schemes are based on present day patterns, it appears that the division of regional assemblages across the tropics is linked to its biogeographic history and the formation of several historical barriers to dispersal (Cowman and Bellwood, 2013a,b). While environmental clines in sea surface temperature are linked to latitudinal variation in diversity (Tittensor et al., 2010), the extensive tectonic, eustatic, climatic, oceanographic and geomorphological (TECOG; Bellwood et al., 2012) processes have played an important role in the origin and maintenance of the tropical biodiversity gradient spanning both deep and shallow times scales (Renema et al., 2008; Pellissier et al., 2014).

The mechanistic processes that underpinned this history and how they have generated such biodiversity has inspired much debate over the last 40 years (Potts, 1985; Briggs, 1999; Bellwood and Meyer, 2009a; Cowman and Bellwood, 2013a) with numerous hypotheses being proposed (Bellwood et al., 2012), but little consensus. The answers to key questions regarding where species have originated and the processes that have promoted speciation and extinction in tropical clades remain unclear. The popularity of phylogenetics, fossil-calibrated age estimation techniques, and the availability of geographic information have allowed biologists to examine the history of the taxa that form the marine biodiversity hotspot. In the case of coral reef fishes and the IAA biodiversity hotspot, what have we learned in the last decade? While new genomic sequencing methods are becoming available (Faircloth et al., 2013) and larger datasets are increasing the resolution of deeper nodes in the Fish Tree of Life (Near et al., 2012), what gaps remain in the evolutionary record of reef associated fishes? How complete is our understanding of the evolution of biodiversity on tropical reefs and which questions will benefit from more sampling, more data, and new analytical approaches?

In this review, I examine the phylogenetic and biogeographic completeness of key families found in reef habitats globally. By exploring the current state of the biogeographic history of tropical reef fishes I highlight where further analysis and discussion is needed, and what new questions require answers.

## EVOLUTION OF FISHES ON CORAL REEFS – FILLING IN THE GAPS

Bellwood and Wainwright (2002) discussed the biogeographic history of fishes on coral reefs. They stated that from the integration of systematics, biogeography, ecology, and paleontology a new understanding of the nature of reef fishes would arise. Twelve years on, the integration of methods and multiple datasets has cast a wide net across the fields of reef fish ecology, evolution and biogeography to give vast insight into the important phases of evolution of coral reef fishes over the past 400 million years (Bellwood et al., in press). A major part of this insight has come from the combination of molecular phylogenetics and the fossil record to form a temporal framework in which to ask questions regarding the origin and tempo of reef fish diversification. Although only a handful of new fossils have been described with reef affinities in the last decade (Carnevale, 2006; Micklich et al., 2009; Bannikov, 2010) the fossil record of early reef fish forms continues to provide a wealth of information on the early origins of reef association in teleost fishes. New analytical techniques have revealed the origins and diversification of anatomical features (Friedman, 2010), important morphological transitions (Goatley et al., 2010), and the emergence of essential functional roles on coral reefs (Bellwood et al., 2014a,b). However, it is the utility of fossils as calibrations points on molecular phylogenies that have allowed the evolution of reef associated lineages to be studied on an absolute timescale. In particular, while the origins of several reef fish groups can be found in the fossil deposits of the Monte Bolca *Lagerstätten* (50 mya; Bellwood, 1996) the crown ages and the diversification of major lineages that lack a fossil record have only been examined with the aid of calibrated chronograms.

There has been debate over what characterizes a ‘coral reef’ fish (Bellwood, 1998; Robertson, 1998), but a general list of ‘reef’ fish families (**Table 1**) identifies those groups that are characteristic of a modern reef assemblage (both coral and rocky reefs), regardless of geographic location (Bellwood and Wainwright, 2002). Indeed, species counts of these families on coral reefs around the world are found in relatively similar proportions (Bellwood and Hughes, 2001). Although these nine fish families found on coral reefs are often used as model groups to address questions regarding diversification on coral reefs, there are at least 35 families of acanthomorph fishes that can be considered ‘reef associated’ (Price et al., 2014). Some of these families are monotypic (e.g., Zanclidae) while other can be entirely reef dwelling but not globally distributed (e.g., Siganidae). Interestingly, the most diverse fish family found on reefs, the Gobiidae, containing over 2000 species, has several lineages confined to coral reefs (Herler et al., 2011), yet remains off the list of traditional reef fish families. Its exclusion from this list may be related to their consistent undersampling in geographic surveys (Ackerman and Bellwood, 2000), made ever more difficult by their cryptic nature and many undescribed species. Nonetheless, this non-traditional reef fish family may provide a good model to study speciation and biodiversity on coral reefs (Rüber et al., 2003; Taylor and Hellberg, 2005). While this review focuses on those nine families classically recognized as reef fish families, other lineages found on (and off) reefs might provide further insight into the evolution of tropical biodiversity. The utility of these families and lineages should be determined by several factors, most importantly, the level at which they have been sampled for phylogenetic reconstruction. As the nine reef fish families are prominent on coral reef around the globe they have been examined with phylogenetic methods and increasing levels of genetic data over the past two decades.

**Table 1:** Diversity, phylogenetic and geographic sampling of the nine characteristic reef fish families.

Family	Richness	% Reef	% *F*	% EToL	% *R*	% *N*	%GASPAR
Chaetodontidae^1^	128	96.88	74.22	10.16	73.44	5.47	96.09
Labridae^1^ (+parrotfishes^2^)	609	83.25	50.41	8.87	39.41	3.28	87.19
Blenniidae^3^	383	44.91	26.63	6.53	10.18	1.04	82.25
Holocentridae^4^	84	80.72	51.19	21.43	22.62	4.76	84.52
Pomacentridae^5^	375	94.4	55.47	9.33	46.13	1.07	94.13
Acanthuridae^6^	81	97.53	77.78	18.52	59.26	6.17	100
Apogonidae^1^	345	72.01	20.87	4.64	6.09	1.16	84.93
Mullidae*	68	48.53	NA	7.35	19.12	4.41	63.24
Carangidae^7^	151	45.03	33.11	15.89	54.97	3.97	65.56

Species richness and percentage of reef associated members are taken from http://www.fishbase.org. % *F* is the percent sampling of species in published family level study; % EToL is the percent sampling of species in the Euteleost Tree of Life (Betancur-R et al., 2013); % *R* is the percent sampling of species in the published phylogeny of Rabosky et al. (2013); % *N* is the percent sampling of species in the published phylogeny of Near et al. (2013); % GASPAR is the percent of family richness that are present in the checklists of the GASPAR database (Parravicini et al., 2013). Superscript denotes source of family level phylogeny: 1-Cowman and Bellwood (2011); 2-Choat et al. (2012); 3-Hundt et al. (2014); 4-Dornburg et al. (in press); 5-Frédérich et al. (2013); 6-Sorenson et al. (2013); 7-Reed et al. (2002). *No phylogeny from a family level study was accessible for the Mullidae.

**FIGURE 2:**
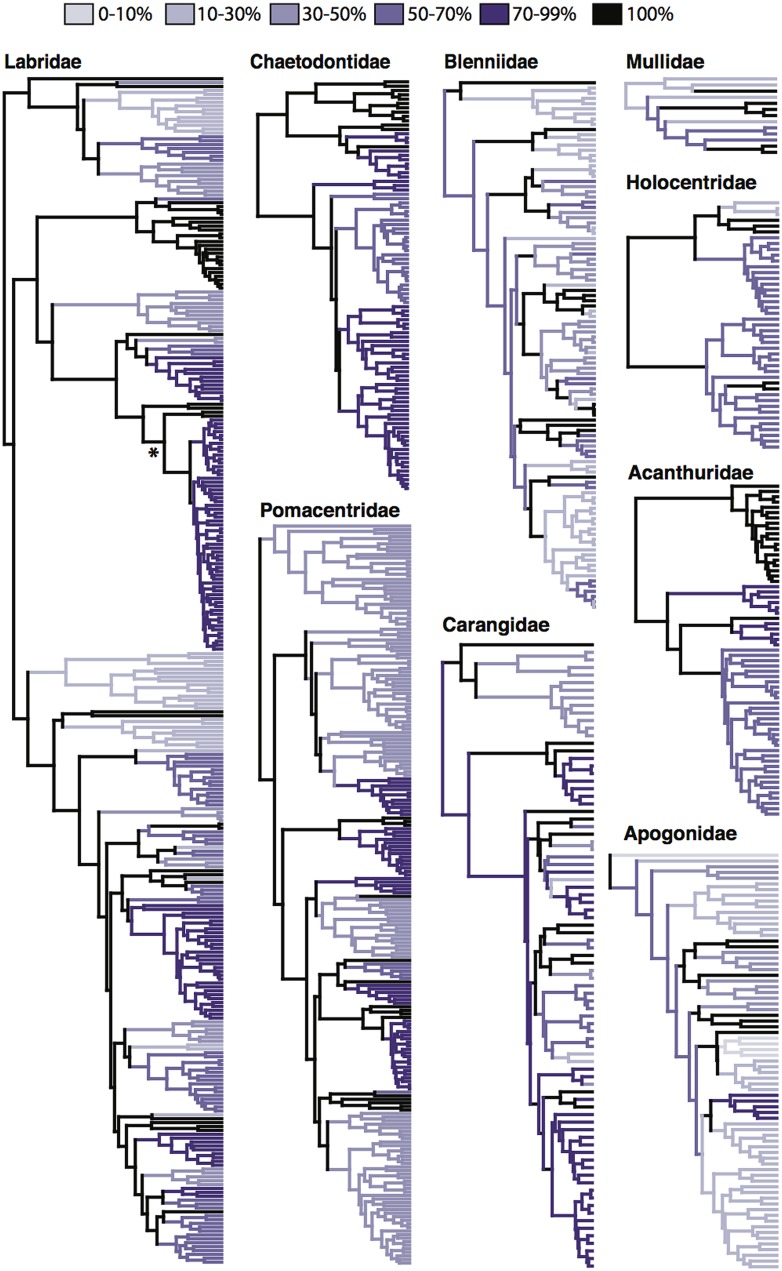
**Phylogenetic sampling of characteristic reef fish families.** Published chronologies of the nine characteristic reef fish families found globally on coral reefs (Bellwood and Wainwright, 2002). Sources of these trees can be found in **Table 1**. Level of taxon sampling per lineage is denoted by color with black branches completely sample. Percent sampling was calculated by a per genus basis with species counts taken from Fishbase (). In the cases of the families Labridae, Chaetodontidae, Pomacentridae and Apogonidae lineage richness estimates were taken from Cowman and Bellwood (2011). Asterisk indicates node where the parrotfish phylogeny of Choat et al. (2012) was grafted to the Labridae tree of Cowman and Bellwood (2011).

Early molecular phylogenetic studies within reef fish groups contained a small number of taxa in select genera (Lacson and Nelson, 1993; McMillan et al., 1999; Bernardi et al., 2001; Reed et al., 2002). Later, the combination of generic level phylogenies with relaxed clock methods (Sanderson, 2003) allowed the estimation of ages of divergence within several reef-associated lineages (Bellwood et al., 2004; Bernardi et al., 2004; Klanten et al., 2004; Barber and Bellwood, 2005; Read et al., 2006). With improved sequencing efforts at the family level (Westneat and Alfaro, 2005; Cooper et al., 2009; Thacker and Roje, 2009), molecular datasets have given insight into the crown origins of reef fish groups and the tempo at which they have diversified (Westneat and Alfaro, 2005; Alfaro et al., 2007; Fessler and Westneat, 2007; Cowman et al., 2009; Frédérich et al., 2013). Even though the characteristic nine families have been the focus of many phylogenetic studies (albeit some more than others), as of yet, not one of these families is represented by a fully sampled, species level phylogeny (**Table 1**). While the majority of major lineages and genera are sampled within these phylogenetic studies, the level at which species within these lineages are sampled varies dramatically (**Figure 2**). Those families that have more completely sampled phylogenies have achieved it through the combination of multiple sequence datasets and the use of supermatrix phylogenetic methods. The combination of datasets for the butterflyfish family Chaetodontidae (Fessler and Westneat, 2007; Bellwood et al., 2010) has resulted in a phylogeny that is over 70% complete (**Table 1**; Cowman and Bellwood, 2011). Similarly, the family Acanthuridae is nearly complete (76%) through the combination of previously published and new sequence data (Sorenson et al., 2013). Other families have been the focus of several phylogenetic studies, incrementally increasing taxon sampling as more data or specimens become available, e.g., the wrasses, family Labridae (now inclusive of odacids and parrotfishes; Westneat and Alfaro, 2005; Alfaro et al., 2009; Cowman et al., 2009; Kazancioglu et al., 2009; Cowman and Bellwood, 2011); and the damselfishes, family Pomacentridae (Cooper et al., 2009; Cowman and Bellwood, 2011; Frédérich et al., 2013). Within the Labridae and Pomacentridae, shallower lineages have also been examined with increased sampling to explore a variety of evolutionary and ecological questions (Smith et al., 2008; Choat et al., 2012; Hodge et al., 2012; Litsios et al., 2012). Other families, such as the Blenniidae and the Apogonidae have been plagued by taxonomic issues that are only beginning to be addressed with more taxa and multi-locus datasets (Thacker and Roje, 2009; Hundt et al., 2014). The incomplete phylogenetic sampling for reef fishes is exacerbated by the rate of new species descriptions and identification of cryptic species (Zapata and Robertson, 2006; Mora et al., 2008; Bowen et al., 2013). Recently, Allen (2014) reviewed the systematics of Indo-Pacific coral reef fishes over the past three decades to reveal that over 1,400 new species have been described with an average of 51.3 new species description per year since 2010.

The incomplete sampling observed in these reef fish families appears to have been a general symptom seen across all fishes when compared to other vertebrate branches of the Tree of Life (Thomson and Shaffer, 2010a). However, there have been three recent efforts in reconstructing the Fish Tree of Life (Betancur-R et al., 2013; Near et al., 2013; Rabosky et al., 2013) with new sequencing methods (Faircloth et al., 2013) providing an exciting avenue for future phylogenomic research in fishes. These ‘top down’ approaches to reconstructing the Fish Tree of Life have greatly improved the resolution of deep nodes and divergences in the major fish groups, including those with reef affinities. These datasets have included varying degrees of taxon sampling of reef associated lineages (**Table 1**), depending on the core aim of the study. The chronogram of Near et al. (2013) concentrated on sampling all families of acanthomorphs with as complete a molecular matrix as possible. While it does not have high species level sampling of reef fish lineages, it has allowed the exploration of rates of transition of fish lineages (at the family level) on and off of reefs over the past 100 million years (Price et al., 2014). The chronogram of Rabosky et al. (2013) closely matches the sampling effort of family level studies of the nine characteristic groups, achieved by mining the published sequence data available on GenBank. In the cases of the families Carangidae and Mullidae, this concatenated super-matrix approach included more species than any other published phylogeny for each family (**Table 1**). These large-scale phylogenetic studies employing supermatrix methods have also allowed the identification of the closest sister families to prominent reef fish families. However, disagreement among these large phylogenies still exists for some families. For example, the closest sister group to the Labridae changes from being the family Centrogenyidae (Betancur-R et al., 2013), to the family Ammodytidae (Rabosky et al., 2013), to the family Gerridae (Near et al., 2013). This highlights the utility of supermatrix approaches, but caution is still needed in their implementation (Thomson and Shaffer, 2010b). In the case of fishes, more work remains to resolve some of these early diverging lineages at the top of the percomorph “bush” (Nelson, 1989), where the origin of several reef associated lineages are found. While these top–down approaches continue to reveal the early evolution of reef fishes, ‘bottom–up’ studies concentrating on the origins of extant lineages have provided a framework to examine the diversification of reef fishes over the past 65 million years.

## DIVERSIFICATION OF FISHES ON TROPICAL REEFS

Phylogenetic sampling and the resolution of reef fish lineages remains a key issue for future research. However, for those groups that have been the focus of age estimation studies, some general, concordant patterns have emerged. The stem lineages of many reef lineages extend back into the Cretaceous (Near et al., 2013) while the crown origins are strongly associated with the aftermath of the K-Pg boundary mass extinction event (∼65 ma; Bellwood et al., in press). A recent study of family level transitions into reef habitat and associated morphological divergence has outlined two waves of colonization before and after the K-Pg boundary (Price et al., 2014). Initial colonization of lineages before the K-Pg boundary (90–72 mya) was accompanied with morphological divergence of clades, while the subsequent wave of reef colonization (65–56 mya) appears to saturate with increasing convergence in morphospace (Price et al., 2014). Patterns of reef invasions within families are likely to be more dynamic (Price et al., 2014) with trophic evolution showing increasing association between fishes and the reef benthos (Cowman et al., 2009; Bellwood et al., 2010). From the appearance of more generalist trophic modes in the Eocene/Oligocene, new and novel trophic modes began to appear on reefs in the Miocene with the trophic system in place by 7 mya (Cowman et al., 2009; Bellwood et al., 2010). Some reef fish lineages have diversified ecologically by expanding into novel areas of morphospace (Friedman, 2010; Price et al., 2011) while others have exhibited convergent radiations across similar trophic strategies (Frédérich et al., 2013). An association with coral reef habitat appears to both promote clade diversity, with higher reef occupancy linked to faster rates of diversification (Alfaro et al., 2007; Cowman and Bellwood, 2011), and increased rates of morphological diversification within lineages (Price et al., 2011, 2013). Lineage diversity and morphological divergence do not appear to be related in these groups (Cowman et al., 2009; Price et al., 2011), however key innovations have been linked to increased diversity in some clades (Kazancioglu et al., 2009; Litsios et al., 2012; Wainwright et al., 2012). In addition, an over arching link between rate of body size evolution and rate of diversification appears to be a general trend across the fish tree of life (Rabosky et al., 2013), but its affect on the evolution of reef clades has not been examined.

By the end of the Eocene, major lineages leading to present day genera and tribes within reef fish families were in place for many reef fish families (Cowman and Bellwood, 2011). After what may have been a cryptic extinction event near the Eocene/Oligocene boundary, coinciding with the origin of the butterflyfishes (∼33 mya), a rebound in cladogenesis within reef associated lineages during the Oligocene/Miocene underpinned much of the extant diversity seen on todays reefs (Cowman and Bellwood, 2011). Several lineages within the Labridae, Pomacentridae, Apogonidae and Chaetodontidae display significantly more diversity than expected, with the most reef-associated lineages appearing more resistant to higher extinction rates than their non-reef counterparts (Cowman and Bellwood, 2011). Elevated cladogenesis has previously been identified in several marine fish lineages (Rüber and Zardoya, 2005), with reef association or habitat shifts suggested to be the underlying mechanism. Later, the relationship between reef association and elevated rates of diversification was demonstrated in Tetraodontiformes (Alfaro et al., 2007), marine gastropods (Williams and Duda, 2008), and more recently in sharks (Sorenson et al., 2014). Whether reef habitats promote this diversity through elevated speciation, or relaxed extinction remains to be seen. As extinction rates are notoriously difficult to estimate from molecular phylogenies in the absence of a paleontological record (Quental and Marshall, 2009, 2010; Rabosky, 2009b), the vital evidence in the form of Miocene fossils for many reef fish lineages, at least, remains out of reach. The expansion of coral reef habitat in the Miocene may have promoted cladogenesis, and provided a refuge from extinction, two processes that may vary on both temporal and geographic scales (Cowman and Bellwood, 2013a).

**FIGURE 3:**
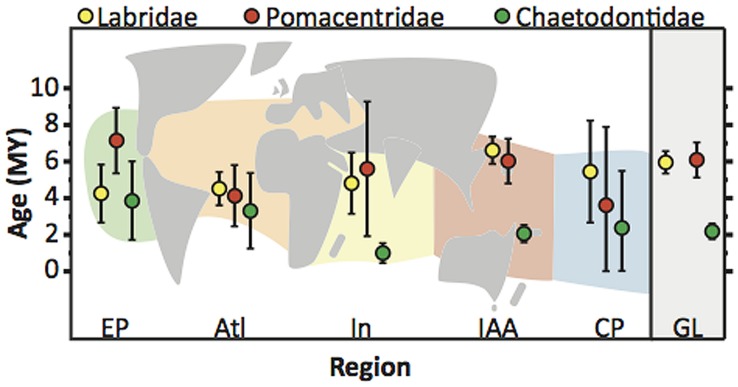
**Biogeographic ages of species of the families Labridae, Pomacentridae, and Chaetodontidae.** Plot shows mean (circle) and 95% CI (whiskers) of the distribution of ages of origination of extant lineages in each biogeographic region, and globally for the Labridae, Pomacentridae, Chaetodontidae (data from Cowman and Bellwood, 2013a). Underlying schematic map shows regional scheme used by Cowman and Bellwood (2013a) for ancestral biogeographic reconstruction. This scheme differs from the regional scheme of Kulbicki et al. (2013) shown in **Figure 1C**. Age distributions for each region represent the ages of extant lineages that originated in that particular region accounting for ancestral biogeographic reconstruction (see Cowman and Bellwood, 2013a). EP, East Pacific; Atl, Atlantic; In, Indian Ocean; IAA, IAA Hotspot; CP, Central Pacific Islands; GL, Global.

The majority of extant coral reef fishes examined by Cowman and Bellwood (2011, 2013a) are of Miocene age (23–5 mya; **Figure 3**) with some possibly being older than the IAA hotspot (Renema et al., 2008). While some geographic variation in the reconstructed ages of lineages exists (**Figure 3**), the older ages of extant species challenged the early suggestion that sea level fluctuations during the Pleistocene was a major factor in the origin of modern coral reef assemblages (Potts, 1985). For reef fishes, the majority of cladogenetic events occurred in the Miocene but speciation still continues in several groups from the Pleistocene onward (Rocha and Bowen, 2008). Pleistocene speciation in these groups may be linked to patterns of barrier vicariance (Bowen et al., 2013; Cowman and Bellwood, 2013b), peripheral budding (Hodge et al., 2012), and more recent fluctuations in coral reef stability (Pellissier et al., 2014). Pleistocene processes may very well have played an active role in the evolution of butterflyfishes (McMillan and Palumbi, 1995), which display younger global ages of extant lineages (∼2.6 mya) than labrids (∼6.7 mya) and pomacentrids (∼6.7 mya), particularly in the Indian Ocean and IAA hotspot (**Figure 3**). It is likely that when the gaps in taxonomic sampling of these reef fish families are filled, and cryptic species are identified, the inclusion of unsampled lineages closer to the present may enhance the role played by speciation in the Pleistocene and the importance of peripheral locations in promoting biodiversity. Processes at work in the Miocene appear to be the main source of origination of modern reef fish biodiversity patterns, while processes maintaining this pattern are prominent from the Pliocene/Pleistocene. However, to gain a clearer picture of the magnitude of these processes across the marine tropics, studies with a biogeographic focus have been important.

## BIOGEOGRAPHY AND BIODIVERSITY

As with the phylogenetic history of reef fishes, the biogeographic history is reliant on sampling, specifically, knowledge of the current extend of reef fish species ranges. In this regard, we are fortunate to have had many skilled ichthyologists throughout the decades collecting geographic information on reef fish distributions (Allen, 2014). Several initiatives have been actively cataloging the diversity found on and off coral reefs, e.g., Atlas of Living Australia^1^; IUCN red list^2^; the Global Biodiversity Information Facility^3^; Map of Life^4^; and Ocean Biogeographic Information System^5^. A recent effort to construct a global database for tropical reef fishes has resulted in over 6300 records for reef fishes across 169 locations (GASPAR database; Kulbicki et al., 2013; Parravicini et al., 2013). This database has been used to explore global predictors of reef fish species richness (Parravicini et al., 2013); global biogeography of reef fishes (Kulbicki et al., 2013); human mediated losses of phylogenetic and functional diversity (D’agata et al., 2014); and the role of stable reef habitat in preserving reef fish diversity (Pellissier et al., 2014).

**FIGURE 4:**
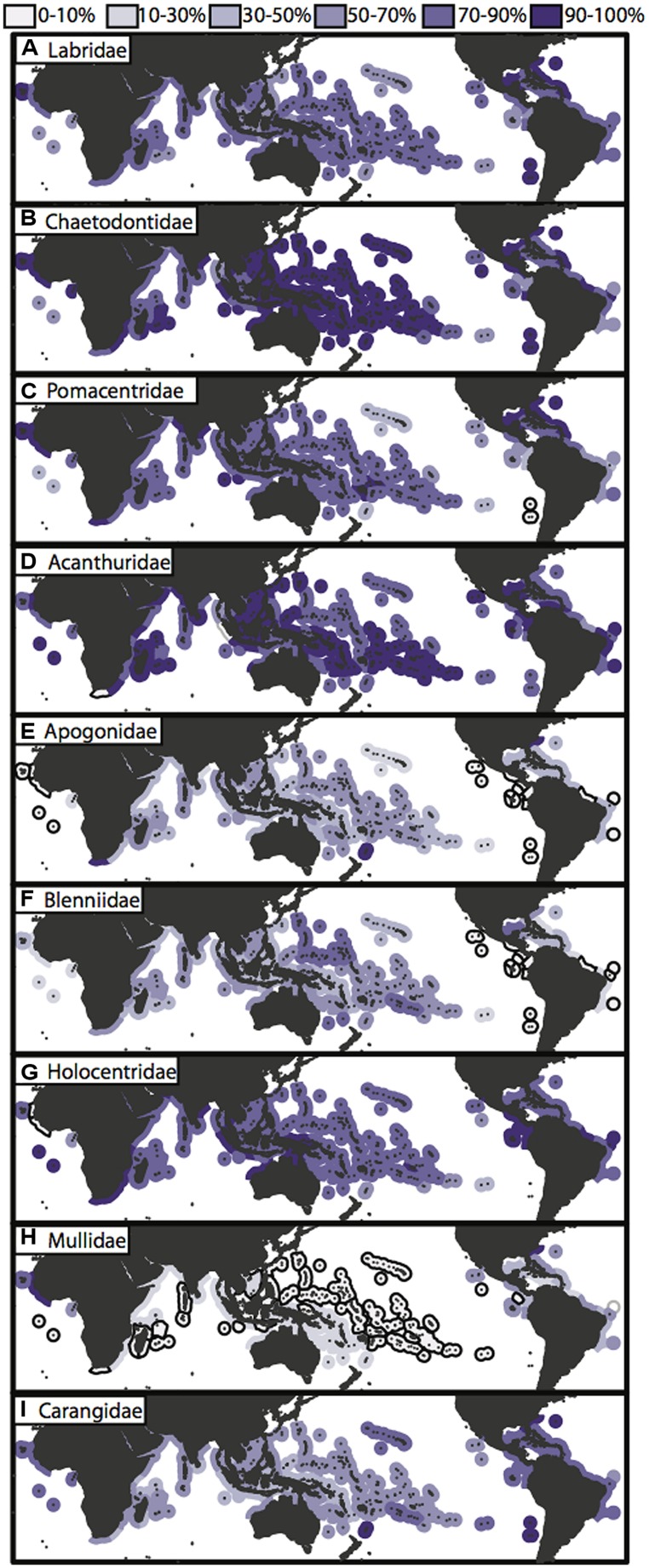
**Phylogenetic sampling of nine reef fish families across the marine tropics. (A–I)** Global maps of tropical ecoregions displaying phylogenetic sampling of species assemblages for each of the nine characteristic reef fish families. Species richness for each family within ecoregions is base on species checklist of species counts from the “checklist” × “all species” dataset of Kulbicki et al. (2013) and phylogenetic sampling is based on taxon sampling of each published family phylogeny (**Figure 2**; **Table 1**). Ecoregions that have <10% of the family species pool represented are outlined in black.

Of the valid nominal species in the nine reef fish families, these geographic checklists (Kulbicki et al., 2013; Parravicini et al., 2013) include the vast majority of them, ranging from 63% of carangid species, to 100% of acanthurid species (**Table 1**). These data are likely to include the majority, if not all reef associated members of these families. In combination with a fully sampled phylogeny of reef fishes, these geographic data would allow us to tease apart some of the questions that have been partially answered so far regarded the origins of tropical biodiversity. Unfortunately, fully sampled phylogenies for important groups are still out of reach. The incomplete and clade biased phylogenetic sampling also translates into a bias in geographic sampling (**Figure 4**). Those charismatic families such as the Labridae and Chaetodontidae that have been the focus of several papers from a variety of research groups have more even phylogenetic sampling across biogeographic ecoregions, with over 50% of taxa present in each region represented in a published phylogeny (**Figures 4A,B**). A sampling bias can be observed among ocean basins, and among families, where some families (Apogonidae, Blenniidae; **Figures 4E,F**) have higher phylogenetic sampling in Indo-Pacific locations, whereas others (Pomacentridae, Mullidae, Carangidae; **Figures 4C,H,I**) show higher phylogenetic sampling in the Atlantic locations. Overall, the families Apogonidae, Blenniidae, Mullidae and Carangidae show concerning levels of lower phylogenetic resolution across tropical reef habitats (**Figures 4E,F,H,I**) with many regions showing below 10% phylogenetic sampling of ecoregion assemblages. Nonetheless, the geographic data available, regardless of its sampling in phylogenetic trees, have been fruitful in delineating biogeographic regions across the marine tropics.

Biogeographic science has an important role in the guidance of biodiversity conservation (Whittaker et al., 2005). Dividing the tropics into discrete regions has proven to be a difficult process (Mouillot et al., 2013), but it is a necessary step toward critically evaluating and implementing conservation priorities (Whiting et al., 2000; Olson et al., 2001). With the predictions of a grim future ahead for coral reef systems under a changing climate (Hughes et al., 2003), the biogeographic delineation of the marine tropics and how regional assemblages have formed through time is paramount to our understanding of biodiversity maintenance. In the past decade, several studies have provided a schematic break down of regions across the tropical belt based on differing criteria (Spalding et al., 2007; Briggs and Bowen, 2012; Kulbicki et al., 2013). Most recently, Kulbicki et al. (2013) used a hierarchical approach to delineating tropical reef regions based on species dissimilarity. Using species checklists across 169 locations (Parravicini et al., 2013), their results identify three realms (Atlantic, Central Indo-Pacific, Tropical East Pacific; **Figure 1C**), each with varying degrees of structure within those delineated regions and provinces (**Figure 1C**; Kulbicki et al., 2013). The Central Indo-Pacific region, within the Indo-Pacific realm, was characterized by lower within region dissimilarity, while neighboring regions (Western Indian and Central Pacific) could be broken down further into provinces (although some internal structure is seen when analyses were based on ecoregions as base units; see Kulbicki et al., 2013). While the IAA (or Coral Triangle) may be delineated as the area containing the highest proportion of reef fish species (Briggs and Bowen, 2012), in terms of species composition there is no strong evidence delineating it as a separate entity in the Central Indo-Pacific (Kulbicki et al., 2013). The IAA biodiversity hotspot may not be a defined region based on species dissimilarity. But, an area the extent of the IAA at the center of the highest number of overlapping species ranges must have played a significant role on an evolutionary scale in the generation of current day biodiversity patterns. On a shallow timescale, the complex role of the IAA and Coral Triangle region has been illustrated through numerous population level and phylogeographic studies (reviews by Carpenter et al., 2011). While the extant ranges of reef associated fishes can statistically delineate regions of dissimilar assemblages, the lines of division are highly dependent on the method used (Leprieur et al., 2012; Mouillot et al., 2013), and there remains no consensus on which method or regional scheme is best. It is likely that the appropriate biogeography scheme will depend on the question being addressed. From a macroevolutionary perspective, whether any present day scheme for biogeographic delineation has a meaning for past diversification and biogeographic evolution has yet to be addressed.

The evolution of reef fish biodiversity patterns is likely to be concordant with the evolution of coral reef habitats. Higher diversification rates of reef associated fish lineages have been demonstrated (Cowman and Bellwood, 2011) and transitions onto coral reefs appear important for accelerated morphological evolution (Price et al., 2014). However, a direct (or indirect) link between the diversification of corals and the diversification of their associated fish lineages has yet to be recognized (Duchene et al., 2013). From a biogeographic perspective, the spatial and temporal distribution of coral taxa and the platforms they construct may provide insight into the evolution of reef fishes that inhabit them. Extent of coral reef area (Bellwood and Hughes, 2001) and its stability through time (Pellissier et al., 2014) have been highlighted as significant predictors of extant reef fish biodiversity. The fossil record of reef building corals highlight differences among ocean basins (Budd, 2000; Wallace and Rosen, 2006). The Atlantic and Caribbean fossil reef biota display high turnover of coral species and extinction of reef habitat (Budd, 2000), while the Indo-Pacific fossil biota displays a history of eastward movement linked to tectonic activity (Wilson and Rosen, 1998; Renema et al., 2008) with modern coral taxa in the Central Indo-Pacific consisting of Tethyan relicts and recent speciation events (Wallace and Rosen, 2006). Such data could be used to model the spatial and temporal dynamics of coral reef habitat allowing us to test more explicit biogeographic scenarios and hypothesis related to tropical biodiversity (e.g., Ree and Sanmartín, 2009).

## THE IAA BIODIVERSITY HOTSPOT – A CENTER OF CONFUSION

Although compositionally the IAA hotspot may not currently present a geographic entity within the Central Indo-Pacific Realm (Kulbicki et al., 2013), the area has historically been recognized as a center of biodiversity in the Indo-Pacific (Ekman, 1953). In an effort to understand the processes that have been important in producing the diversity pattern across the Indo-Pacific and the associated center of high diversity, three hypotheses became popular in the early 1980s, originally formulated to explain the biodiversity of reef building corals (summerized by Potts, 1985). These ‘center of’ hypotheses have been co-opted in the context of reef fish biodiversity. They have been expanded and modified to explain the extensive and overlapping widespread ranges seen in several reef fish groups (Hughes et al., 2002; Connolly et al., 2003). The details of each of these, and other hypotheses have been reviewed by Bellwood et al. (2012). Both phylogenetic and population level studies of reef fish taxa have highlighted evidence describing the IAA (or the Coral Triangle) as a center of origin (Briggs, 2003; Timm and Kochzius, 2008), a center of overlap (Hubert et al., 2012; Gaither and Rocha, 2013), or a center of accumulation/survival (Barber and Bellwood, 2005; Kool et al., 2011).

Each hypothesis has made predictions about the location of origin of species, their age, and their trajectory of range expansion or change (see Bellwood et al., 2012). Primarily, species with restricted endemic ranges have been important in the assessment of these hypotheses, but even the study of endemic taxa has been fraught with debate (Bellwood and Meyer, 2009a,b; Briggs, 2009). Even how an endemic range is defined can lead to conflicting patterns of endemism (Hughes et al., 2002; Mora et al., 2003). Bellwood and Meyer (2009a,b) highlighted the diffuse ages of endemic taxa, whether they are found inside or outside the IAA. Endemic taxa can be young (neo-endemics) or old (palaeo-endemics) and as such their use to delineate areas of species geographic origin should be cautious. Indeed the ages of endemic coral reef fishes in several families do not differ significantly from those of more widespread species (Hodge et al., 2014). Instead of using endemic species as a tool in pinpointing locations of species origin it is becoming clear that understanding how processes of isolation and extinction have lead to current patterns of endemism along side widespread species is an important step in the study of reef fish biodiversity.

Recent studies have begun to highlight that the processes that promote, maintain and diffuse biodiversity in the marine tropics are more dynamic in nature with multiple drivers acting both in concert, and decoupled across temporal and geographic scales (Bowen et al., 2013; Cowman and Bellwood, 2013a). The question has changed from which hypothesis is most accurate, to when and where the processes they invoke have been most prevalent and how they have interacted to produce the biodiversity we see today. To this end, it may be time to mute the discussion about ‘centers of’ in the field of reef fish biodiversity in favor of directly examining and modeling rates of speciation, extinction and dispersal in a temporal and geographic framework. Such methods have been advantageous in investigating terrestrial diversity patterns on global scales (Jetz et al., 2012; Rolland et al., 2014). If these different processes have played an active role in the development of tropical biodiversity but on different temporal and spatial scales, then several biogeographic areas may have historically acted as sources or sinks (or both) for biodiversity at different periods in time. For example, the Atlantic realm, like the IAA hotspot, can be considered a center for species origination (Cowman and Bellwood, 2013a), but its history of isolation from the Indo-Pacific (Floeter et al., 2001; Joyeux et al., 2001) and extinction (O’Dea et al., 2007) has contributed to its lower diversity when compared to the Indo-Pacific. Both the Indian Ocean and the Central Pacific regions have higher standing diversity of reef fishes than the Atlantic. However, most of their diversity has been derived through expansions of lineages from the IAA. But peripheral locations in both these regions have also been sites of species origination (Hodge et al., 2013). In addition, within the Indo-Pacific realm, it remains unclear if the IAA hotspot actually has experienced higher rates of speciation than adjacent regions (Bellwood and Meyer, 2009b; Litsios et al., 2014). While speciation has certainly occurred within the IAA hotspot, peripheral locations are also important sources of new species (Bowen et al., 2013; Hodge et al., 2014). None of these hypotheses can be disregarded, but nor can any one of them solely explain the IAA biodiversity pattern (Rosen, 1984; Palumbi, 1997; Halas and Winterbottom, 2009; Hoeksema, 2009). Halas and Winterbottom (2009), comparing reconstructed area relationships of cladograms of fishes, corals and molluscs, found little congruence among these taxa and little evidence for any of the core models examined, despite these groups displaying very similar patterns of diversity across the tropics (Roberts et al., 2008). Several studies have asked what present day geographic or environmental factors explain the variation in the IAA diversity pattern (Mora et al., 2003; Tittensor et al., 2010; Parravicini et al., 2013), but it appears that examining historical factors may have more explanatory power when examining the origin and maintenance of biodiversity in the marine tropics (Renema et al., 2008; Pellissier et al., 2014). While the history of tropical biodiversity may remain clouded until complete phylogenies are available, concordant patterns in currently published data for tropical reef fishes has allowed key events in the history of the tropics to be recognized.

Though the IAA hotspot is enigmatic, it has not been a unique pattern through time. It represents the modern manifestation of a pattern that has existed for at least the past 50 million years (Renema et al., 2008). The center of biodiversity has ‘hopped’ from a Tethyan location (Paleocene), to an Arabian/IAA hotspot (Eocene/Oligocene), to its current location in the IAA (Miocene; Renema et al., 2008). This biogeographic re-centering of biodiversity was associated with a sequence of TECOG events (Bellwood et al., 2012), dynamic processes controlling the origin and survival of species (Cowman and Bellwood, 2011, 2013a) resulting in the establishment of a trophic system characteristic of modern coral reefs. These processes resulted in the contraction and expansion of carbonate platforms, the evolution of the coral species that built them, and their associated fish lineages.

The earliest fossil records of lineages leading to modern coral reef fishes and the coral genus *Acropora* are found in close proximity in the Late Paleocene/Early Eocene deposits of Europe and the Western Indian Ocean (Bellwood, 1996; Wallace and Rosen, 2006). These deposits can be realistically extrapolated to be associated with the ancestral hotspot centered in the Western Tethys seaway (Renema et al., 2008). No fossil *Acropora* are currently know from the Eocene of the Indo-West Pacific. While this could be an observational artifact, this gap in the coral record corresponds to a geographic gap with fewer shallow water habitat for coral growth in the Indo-Pacific at that time (Wilson and Rosen, 1998). It is not until the Late Oligocene/Early Miocene (∼26 mya) where we see the first fossil evidence of coral species of the genus *Acropora* occurring in the IAA (Wallace and Rosen, 2006). From this time, the tectonic collision of Australian and South East Asian plate fragments favored localized isolation and origination of new coral taxa and the expansion of carbonate platforms in the IAA (Wilson and Rosen, 1998). It is during this time we also see the demise of carbonate platforms in Europe and the Mediterranean deposits (Wallace and Rosen, 2006) and the collapse of the ancestral Tethyan and Arabian biodiversity hotspots (Renema et al., 2008). This collapse of the ancestral hotspots is associated with an eastward shift in fossil deposits of reef associated organisms (Renema et al., 2008) and the expansion of carbonate platforms in the IAA (Wallace and Rosen, 2006). A period of high extinction may be visible in the molecular record of some reef fish groups coinciding with a decrease in fossil numbers of all marine taxa (Cowman and Bellwood, 2011). More fossil data for focal fish groups is required to confirm this pattern, however, a total evidence approach including fossil taxa as dated tips in an ancestral biogeographic framework for the family Holocentridae holds promising insight (Dornburg et al., in press).

The Miocene epoch represents an important phase in the evolution of the IAA biodiversity hotspot (Bellwood et al., in press), with the expansion of both coral reef platforms (Wallace and Rosen, 2006) and associated fish lineages (Cowman and Bellwood, 2013a). As a result of tectonic activity we see the final closure of the Tethys seaway, known as the Terminal Tethyan Event (TTE, 18–12 mya; Steininger and Rögl, 1979) and the development of the Isthmus of Panama (IOP; Coates and Obando, 1996) isolating the Atlantic and Caribbean from the Indo-Pacific. The development and closure of these ‘hard’ land barriers would have been associated with climatic upheaval (Hallam, 1994; Montes et al., 2012) and extinction in reef locations (McCoy and Heck, 1976; Budd, 2000; O’Dea et al., 2007). This has led to a diffuse pattern of vicariance in the molecular record of some reef fish families (Cowman and Bellwood, 2013b). The TTE and the IOP barriers in conjunction with the expanse of ocean known as the East Pacific Barrier (EPB; Bellwood and Wainwright, 2002) have left a lasting mark on modern tropical reef fish assemblages (Kulbicki et al., 2013). However, some recent dispersal from the Indian Ocean into the Atlantic has been detected (Rocha et al., 2005a; Bowen et al., 2006), with several lineages maintaining gene flow across the EPB (Lessios and Robertson, 2006).

In terms of coral reef ecology, the Miocene holds the origins of many novel feeding modes (Cowman et al., 2009; Bellwood et al., 2010), and an escalation in herbivory and detritivory that have become essential services performed by fishes on healthy coral reefs (Hughes et al., 2011). In the Labridae, coral reef associated lineages show significantly higher rates of trophic ecomorphological evolution with over a third of that diversity seen within trophic modes only found on coral reefs (Price et al., 2011). A switch to consuming low quality food items has been linked to higher rates of diversification in several coral reef lineages with origins in the Oligo-Miocene (Lobato et al., 2014). This reflects fossil evidence showing the transition of reef fish forms to exploiting the epilithic algal matrix, an underutilized resource on coral reef flats (Bellwood et al., 2014a).

By the end of the Miocene, the center of fish diversity has taken shape in the IAA (Cowman and Bellwood, 2013a) and important trophic components are in place on coral reefs (Cowman et al., 2009; Price et al., 2011; Bellwood et al., 2014a). Speciation continues in several lineages from the Pliocene to Recent time periods. Expansion of lineage ranges from the IAA to adjacent regions is common (Cowman and Bellwood, 2013a) with vicariance and speciation in peripheral locations (Hodge et al., 2014). In the Atlantic realm, Pliocene speciation has been described in several reef associated genera (Floeter et al., 2008), with evidence of ecological speciation (Rocha et al., 2005b). While ecological speciation is likely to be ongoing in the Indo-Pacific, recent studies reflect a complex history of sympatric, allopatric and parapatric speciation (Rocha and Bowen, 2008; Choat et al., 2012; Hodge et al., 2012, 2013) with rapid dispersal potential (Quenouille et al., 2011) blurring the geographic history of speciation.

## MACROEVOLUTION AND MACROECOLOGY ON TROPICAL REEFS

Albeit incomplete, dated phylogenies combined with biogeographic distributions can detect the initial origins of ancestral reef fish lineages, their extinction and survival with shifting centers of biodiversity, and proliferation within expanding habitat. From these patterns it is possible to identify temporal and spatial variation in rates of speciation, extinction and dispersal and how this variation has resulted in the current biodiversity gradient. Measuring the net rate of diversification and how it varies through time has become an important metric in the integrated study of macroevolution and macroecology (Rabosky, 2009a). Methods to model variation in diversification rates in light of ecological processes has seen dramatic advancement in the last decade (reviewed by Morlon, 2014). In particular, recent interest and debate has grown around whether diversity in clades or assemblages can increase unbounded or if it can be limited by ecological or other factors (Rabosky, 2009a; Morlon et al., 2010). Only a handful of studies have explicitly examined rate variation in reef fish lineages with the comparison of constant and rate variable models of diversification (Rüber and Zardoya, 2005; Alfaro et al., 2007, 2009; Cowman and Bellwood, 2013a; Litsios et al., 2014; Lobato et al., 2014), but none have considered the effects of ecological or other factors in limiting biodiversity among tropical regions. If limiting factors do govern the capacity for biodiversity in clades and communities, variation in tropical reef fish biodiversity may have little to do with rates of speciation or extinction and more to do with the capacity of regions to support biodiversity. Rather, clades or communities have experienced different phases in their rate of diversification where they initial radiate and then slowdown as a limit is approached (Rabosky, 2009a). Variation in where and when clades have radiated would led to the observed patterns in tropical biodiversity. If clades have varied in the timing of their radiating phase among geographic regions this might manifest itself as differences in the ages of lineage origination among regions. This may be the case for some families where data is available (**Figure 3**), however these data are still from incompletely sampled phylogenies.

This radiation and subsequent slowdown is also termed “density-dependent” diversification and can resemble a “niche-filling” process. Such a process was recently uncovered in the trophic diversification of several tropical reef fish families (Lobato et al., 2014) where a switch to low quality food items by several lineages resulted in significant diversification. This highlights the potential for ecological opportunity on reefs to shape lineage diversification. However, it is unclear if this potential has manifested as an actual limit on diversification as many reef fish lineages do not display a slowdown in diversification rate toward the present (Cowman and Bellwood, 2011). While there is evidence of speciation rates decaying over time it appears that limits of diversity in several groups have yet to be realized (Morlon et al., 2010).

We have yet to definitively identify within a complete phylogenetic framework how rates of net diversification on tropical reefs have been altered by ecological or biogeographical processes. If such processes have underpinned the radiation of fishes on coral reefs it may change our understanding of the origins of biodiversity and what factors are important in maintaining diversity in the present.

## TROPICAL BIODIVERSITY AND RATES OF MOLECULAR EVOLUTION

Across several taxonomic groups there is consistent evidence of a link between the rate of molecular evolution and the observed biodiversity of clades (Fontanillas et al., 2007; Lanfear et al., 2010b; Duchene and Bromham, 2013). This pattern is not universal (Goldie et al., 2011) and has yet to be critically evaluated across the Fish Tree of Life. However, a recent study of genomic variation in African cichlids highlights several molecular mechanisms that may be linked to the enigmatic and rapid diversification of the group (Brawand et al., 2014). In the context of biodiversity patterns there is a tangle web among ecological traits, diversification and molecular rate (Dowle et al., 2013). There are a large number of characteristics, ecological and environmental, that can potentially shape the rate at which genes evolve with numerous hypotheses put forward (Bromham, 2011).

When exploring the link between molecular rate and diversity there are three main explanations that have been discussed (Barraclough and Savolainen, 2001). First, there is something about the process of speciation itself that increases the rate of molecular evolution (Venditti and Pagel, 2010). If the rate of speciation is associated with the rate at which populations divide or become isolated, then a reduction in the effective population size could increase the rate of substitution of nearly neutral mutations (Bromham, 2011). Second, the direction of causation could be the opposite where changes at the genomic level drive rates of speciation, and as such directly influence macroevolutionary patterns (Bromham, 2011). Higher mutation rates would result in faster accumulation of incompatibilities among hybrids and hasten the reproductive isolation among populations. Lastly, the association between diversity and molecular rate could be indirect, where a third factor promotes an increase in the rate of molecular evolution and the diversification rate. Methods are available for testing these scenarios (reviewed by Lanfear et al., 2010b) and results tend to show evidence for the rate of mutation influencing diversification (Lancaster, 2010; Lanfear et al., 2010a; Duchene and Bromham, 2013). These hypotheses have yet to be examined in fishes and may provide insight into the underlying mechanics of speciation on coral reefs.

If speciation drives the rate of molecular evolution through population subdivision, higher diversity and molecular rates in reef associated fish lineages could be driven by the fragmentation of habitat and peripheral isolation, both process that have been reported in evolutionary studies of tropical reef fishes (Hodge et al., 2012; Pellissier et al., 2014). If correct, endemic range species should show faster rates of molecular evolution when compared with a widespread sister lineage. Endemic range species and isolated populations within widespread species have displayed increased genetic structure and haplotype diversity than their widespread counterparts (Hobbs et al., 2013). Whether this is a reflection of a fast molecular rate remains to be seen.

If mutation rate drives speciation rate, would this mean that coral reefs provide the molecular fuel for speciation? It has already been demonstrated that coral reefs promote both the diversification of lineages (Alfaro et al., 2007; Cowman and Bellwood, 2011; Sorenson et al., 2014) and morphological diversity (Price et al., 2011, 2013) so it is not unrealistic that they would also speed molecular evolution. But not all lineages found on coral reefs are morphologically diverse, nor are they all biodiverse. If a similar pattern is found in the molecular rates of coral reef fish clades, where only some lineages identify with faster rates, their proliferation may be due to intrinsically higher mutation rates. This higher rate would allow populations that are briefly or partially separated by any number of mechanisms to become reproductively isolated faster. The IAA hotspot may be exceptionally diverse because its complex series of archipelagos and shallow basins provide more opportunity for population separation than elsewhere. A higher mutation rate could also provide more genomic variation for selection to act upon (Bromham, 2011) for adaptation and separation along ecological axis (Schluter and Conte, 2009). Ecological speciation has been documenting on coral reefs (Rocha et al., 2005b), and a link between adaptations to new niches and high diversity (Lobato et al., 2014). For this scenario, there would not be anything particularly special about coral reef association other than it enabling those lineages with higher mutation rates to promote lineage diversification. The IAA being at the center of the diversity gradient would be a consequence of more reef habitat, which has previously been shown as a significant predictor of variation in reef fish diversity (Bellwood and Hughes, 2001). A situation where coral reefs have acted as a medium for the direct influence of molecular rate on diversification is very different from a third scenario where an indirect factor associated with coral reef habitats promotes faster molecular rates, and independently higher diversification. In comparing reef and non-reef habitats, or tropical versus temperate latitudes, there are a number of indirect factors that could promote both molecular rate and diversification (Dowle et al., 2013). However, across the tropical belt it may be difficult to deduce what particular factors mediate the link on coral reefs in the IAA and not on reef in other regions. As with models of diversification, it is likely that when these hypotheses are examined in depth, the processes at play will be more dynamic and possibly include more than one explanation.

## CONCLUSION

In reviewing the current state of phylogenies and historical biogeography of tropical reef fishes I have summarized a series of historical events that have underpinned the origins and proliferation of reef fish biodiversity in the tropics. This review also highlights several groups that require increased sampling and further analysis. While some focal groups are almost completely sampled, an additional push is needed to obtain complete species level sampling. Although the traditional nine coral reef fish families have been important models in the exploration of marine speciation and evolution on coral reefs, there are other fish families and lineages that may provided as much, if not more insight into the origins of tropical biodiversity. It is in this respect that a robust and well-resolved Fish Tree of Life will be beneficial to both the examination and comparison of evolutionary rates among discrete tropical clades found on and off reefs, and the investigation of overarching patterns of tropical diversification. I suggest that future research concerning the macroevolutionary patterns of fishes found on coral reefs examine the historical variation in rates of speciation, extinction, and dispersal among biogeographic regions and across multiple lineages. Further discussion is needed to evaluate how hypotheses concerning the origin and maintenance of biodiversity are modeled to account for the interaction between macroecology, macroevolution and molecular processes.

There are several questions that offer exciting pathways for future research:

• How has temporal and spatial variation in rates of speciation, extinction and dispersal lead to present day patterns of tropic fish biodiversity?
• Do present day biogeographic delineations reflect the evolutionary history of the tropical belt, and which scheme is best?
• Are there limits to the biodiversity of tropical regions and if so, how are these limits linked to the diversity of clades and regional assemblages?
• Do tropical clades experience increase rates of molecular change on coral reefs and how does this link to patterns of biodiversity across the tropical belt?

With increasing access to genomic methods, there is a unique opportunity to reconstruct the evolutionary history of all fishes to the level of resolution that is available in other vertebrate clades. Within this framework we can move beyond categorizing patterns and predictors of extant biodiversity, and statistically examine the evolutionary history under hypotheses driven models of diversification.

## Conflict of Interest Statement

The author declares that the research was conducted in the absence of any commercial or financial relationships that could be construed as a potential conflict of interest.

## Footnotes

1 http://www.ala.org.au

2 http://www.iucnredlist.org

3 http://www.gbif.org

4 http://www.mol.org

5 http://www.iobis.org
