# Corrigendum: Historical factors that have shaped the evolution of tropical reef fishes: a review of phylogenies, biogeography, and remaining questions

**DOI:** 10.3389/fgene.2015.00295

**Published:** 2015-09-15

**Authors:** Peter F. Cowman

**Affiliations:** Department of Ecology and Evolutionary Biology, Yale UniversityNew Haven, CT, USA

**Keywords:** coral reef fishes, ancestral biogeography, marine tropics, phylogeny, diversification

Figure 1A of this manuscript does not have the correct scaling for the distribution of species richness. The correct break values for colors denoting levels of species richness across tropical areas should be: <250, ≥500, ≥1000, ≥1500, ≥2000. This mistake, while not crucial to the discussion is inaccurate based on the dataset examined in this study. A new figure has been generated with correct values. The figure caption remains the same, and there are no associated changes to be made in the main text.

**Figure 1:**
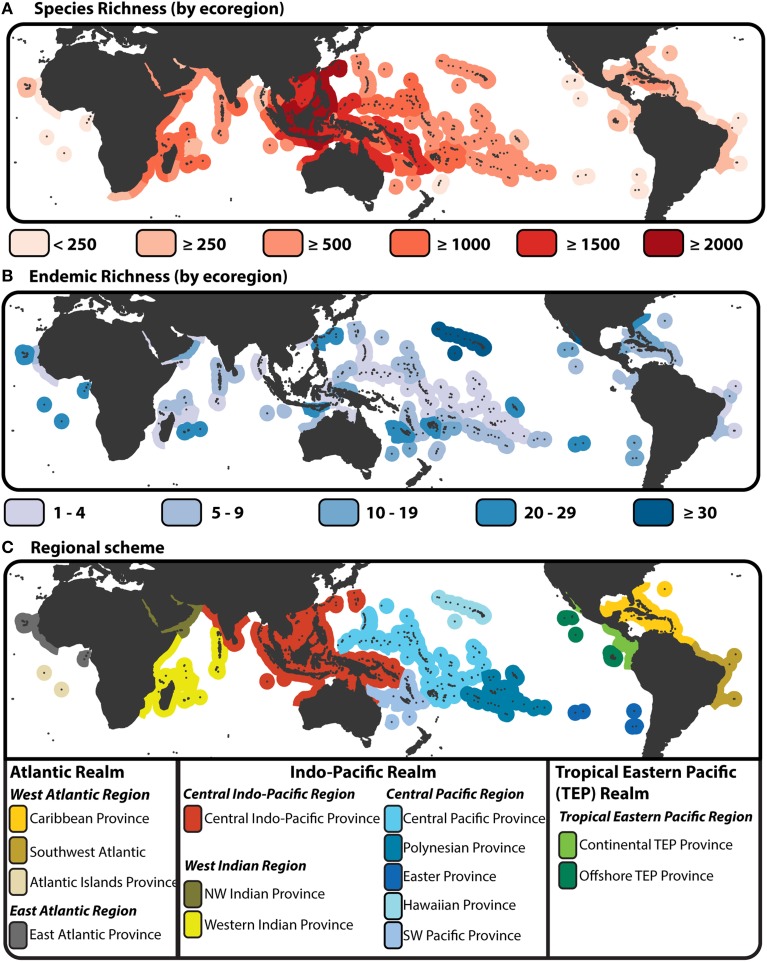
**Species richness, endemism and provinciality of tropical reef fishes**. **(A)** Map of species biodiversity by tropical ecoregion (Spalding et al., 2007) with color gradient denoted areas of high species richness (dark red) to areas of low species richness (light red). **(B)** Map of endemic species by ecoregion. Under this scheme a species is endemic if it is only found in a single ecoregion, i.e., a regional assessment of endemic rather that designated by percent of area comparison (Hughes et al., 2002). Species richness and endemic estimates are based on species counts from the “checklist” × “all species” dataset of Kulbicki et al. (2013). **(C)** Biogeographic delineation of tropical Realms, Regions and Provinces based on species dissimilarity analysis of Kulbicki et al. (2013). This biogeographic scheme is based on checklists as base units (see Kulbicki et al., 2013), however here the scheme is imposed onto of the tropical ecoregions of Spalding et al. (2007).

The original article was updated.

## Conflict of interest statement

The author declares that the research was conducted in the absence of any commercial or financial relationships that could be construed as a potential conflict of interest.
